# Emerging mechanisms and novel targets in allergic inflammation and asthma

**DOI:** 10.1186/s13073-017-0501-6

**Published:** 2017-12-04

**Authors:** Scott T. Weiss

**Affiliations:** Harvard Medical School and Channing Division of Network Medicine, Brigham and Women’s Hospital, Boston, MA 02115 USA

## Abstract

Airway inflammation is key to the severity and persistence of asthma. Recent studies have revealed novel immune mechanisms that target dendritic cells, T helper 2 cytokines, regulatory T cells, and type 2 innate lymphoid cells in allergic inflammation, as well as novel approaches that target airway smooth muscle in asthma. These advances inform the development of new targeted treatments for allergic inflammation and asthma with the potential to provide therapeutic benefit.

## Asthma and allergic inflammation

Asthma is defined by airway hyper-responsiveness and inflammation. The disorder is syndromic, without a clear defining feature such that some are reluctant to call it one disease. Epidemiologic studies suggest that the prevalence of severe asthma in the USA is approximately 8% and the disorder costs the US healthcare system more than 12 billion US dollars per year in hospitalizations, emergency room visits, and days lost from work and school [1]. In addition, the disease can progress to the development of chronic obstructive lung disease or chronic obstructive pulmonary disease.

On-going allergic inflammation is one of the key reasons for asthma persistence and severity.

In mild-to-moderate asthma, the predominant inflammatory response in the airway is T helper 2 (Th2)-type inflammation (also known as type 2 (T2) inflammation), which is driven by CD4+ helper T cells that express the cytokines IL-13, IL-4, and IL-5 and is associated with elevated levels of immunoglobulin E (IgE) and eosinophils in the airways. In more severe asthma, several inflammatory phenotypes exist, often simultaneously. These include the previously mentioned T2 inflammation, but also Th17 inflammation, which is a neutrophilic inflammatory response and, in a small number of cases, a pauci-granulocytic type of inflammation with neither eosinophils nor neutrophils. This latter type of inflammation may not be subject to immune control. T2 inflammation is present in all three types of asthma (mild, moderate, and severe). Th17 inflammation can also be seen in either moderate or severe asthma, and pauci-granulocytic inflammation is only seen in severe asthma [2].

Traditional treatment for mild intermittent asthma is an as-needed, short-acting, beta-2 adrenergic receptor agonist (beta-2 agonist). Beta-2 agonists are the largest class of drugs used to treat asthma, but have remained controversial due to poor clinical responses and potentially life-threatening adverse effects. For mild persistent disease, inhaled corticosteroids with a short-acting beta agonist are used for treatment. For moderate and severe disease, inhaled corticosteroids are combined with a long-acting beta-2 agonist. Patients with the most severe disease may require oral steroids on a regular basis and all patients may need oral steroids during an exacerbation. Patients with mild disease tend not to take their medications as prescribed, often using them only when they have symptoms, rather than to prevent symptoms. Patients with more severe disease regularly take their prescribed medications but often do not get symptom relief. Clearly, new medicines for asthma need to be developed.

Recently, there has been a proliferation of studies that have revealed the mechanisms underlying allergic inflammation and asthma, and are providing possible novel approaches for treatment. These novel mechanisms target dendritic cells (DCs), type 2 innate lymphoid cells (ILC2s), regulatory T (Treg) cells, and airway smooth muscle, or, potentially, all of these. Below, I discuss current immune therapies for asthma, emerging mechanisms and targets for the treatment of allergic inflammation and asthma, and the implications and challenges for medicine.

## Currently available immune therapies: advantages and limitations

The involvement of the vitamin D receptor in asthma susceptibility was identified in linkage and fine-mapping studies. Vitamin D is a potent immune modulator, fine-tuning the immune system to respond appropriately to allergic inflammation. By downregulating the function of DCs and upregulating Treg cells, vitamin D acts as a rheostat on the immune response. The vitamin D receptor is also expressed on airway smooth muscle where higher concentrations of vitamin D relax the smooth muscle and prevent its proliferation [3]. Vitamin D also upregulates the absorption of inhaled corticosteroid across the airway epithelium [4]. Clinical trial data unequivocally support the use of vitamin D in childhood asthma, and one study estimated that the effect was equivalent to that of inhaled corticosteroids, in terms of reducing asthma exacerbations, as measured by hospitalizations and emergency room visits [5].

Monoclonal antibodies to IL-5 and to a combination of IL-4 and IL-13 have been developed. These drugs reduce eosinophil numbers, reduce oral steroid use, and reduce asthma exacerbations in severe asthma [2]. Monoclonal antibodies have also been developed to another type 2 cytokine, thymic stromal lymphopoietin (TSLP), which was implicated in asthma using genome-wide association studies (GWAS), and these TSLP monoclonal antibodies were recently tested in a clinical trial [6]. Additional monoclonal antibodies targeting both the innate and the adaptive immune pathways are currently in development. These drugs are very expensive but are now available for the treatment of severe asthma, which represents 3–10% of the population of adults with asthma. The application of vitamin D or monoclonal antibodies in clinical practice has been minimal due to questions about efficacy (vitamin D) and cost (monoclonal antibodies).

## Novel mechanisms and targets for allergic inflammation

Since the *IL33* gene was implicated in asthma via GWAS, its immunologic function as an alarmin cytokine has been defined. Much work has gone into determining how alarmin cytokines (IL-25 and IL-33) activate ILC2 cells [7]. ILC2 cells are not only involved in mucosal homeostasis but are also involved in the initiation of inflammatory responses. Wallrapp and colleagues recently showed that the neuropeptide receptor gene *Nmur1* was highly expressed in murine ILC2 cells, and that, after IL-25 stimulation, the ligand of NMUR1, neuromedin U (NMU), activated ILC2 cells in vitro and in vivo [8]. Co-administration of NMU and IL-25 strongly enhanced allergic inflammation in vivo [8]. One concern, as with many of these immune mechanisms, is whether blocking this response would have untoward effects on infection. Clearly, more investigation is needed as this response is seen in T2 asthma and so might be a viable pathway to the treatment of the disease, although a drug is still a long-term prospect.

There have also been advances in understanding the regulation of antigen-presenting cells in allergic inflammation. Mechanistic target of rapamycin (mTOR) is a serine/threonine protein kinase that regulates DC function by different mechanisms depending on the type of DC. mTOR promotes type 1 interferon production in plasmacytoid DCs that express CD103, and reduces pro-inflammatory cytokine production by classical DCs that express CD11b [9]. As with vitamin D, there are a variety of other immune processes influenced by mTOR, such as the control of effector T cell number, B cell response, and potentially other immune processes such as those involved in allergic inflammation. Sinclair and co-workers recently showed that mTOR regulated both metabolism and accumulation of CD103+ DCs (plasmacytoid DCs) and alveolar macrophages in the lung in mouse models [9]. Although the numbers of mTOR-deficient CD11b + DCs (classical DCs) in the lung were not altered, these cells were metabolically reprogrammed to shift the balance from eosinophilic Th2 inflammation to a neutrophilic Th17 response. This cellular reprograming was shown to be dependent on classical DCs that both produced IL-23 and increased fatty acid oxidation, both of which are allergic inflammatory markers [9]. These findings suggest that targeting classical DCs might be useful for the treatment of allergic inflammation, but once again further studies are needed before a drug can be developed.

The beta-2 adrenergic receptors are G protein-coupled receptors (GPCRs). A specific type of GPCR, the Gq-coupled receptor, is important in controlling airway muscle tone. Recently, inhibitors of the Gq protein, such as FR900359, have been developed and have been tested in mouse, pig, and human airway tissue [9]. FR900359 prevented bronchoconstriction without effects on blood pressure and heart rate [10]. Further testing of FR900359 and other Gq receptor blockers may reveal these inhibitors to be the first new powerful bronchodilators for many years. These preliminary studies need to be followed up by phase I clinical trials and toxicity profiling, so we are still many years away from having these novel Gq protein inhibitors in the clinic.

## Implications for medicine: challenges and future directions

Genomic discoveries have led to a new understanding of allergic inflammation, and these new approaches and targets for the treatment of asthma and allergic inflammation have great potential. However, there are also concerns. First, the cost to the consumer of novel immune therapies is likely to be high. This is certainly true for monoclonal antibodies. Second, and related to the cost issue, do we really know enough about who is likely to respond to these immune therapies? Asthma sub-phenotyping is still relatively primitive, with mild, moderate, and severe disease classifications, and we need improved application of molecular phenotyping as it relates to specific drug treatment responses. For example, vitamin D seems to work better for children than adults and for those with a milder form of the disease. While those with severe asthma represent a disproportionate share of the care burden, we still need to understand the utility of novel treatments not only for those with severe disease but also for those with mild-to-moderate disease. Ultimately, to truly advance precision medicine for allergic inflammation and asthma we need to be able to deliver these new immune therapies to greater numbers of patients at a reduced cost.

## Abbreviations

DC: Dendritic cell
ILC2: Type 2 innate lymphoid cell

## References

[1] Weiss KB, Sullivan SD, Little CS (2000). Trends in the cost of illness for asthma in the United States, 1985-1994. J Allergy Clin Immunol.

[2] Israel E, Reddel HK (2017). Severe and difficult-to-treat asthma in adults. N Engl J Med.

[3] Himes BE, Koziol-White C, Johnson M, Nikolos C, Jester W, Klanderman B (2015). Vitamin D modulates expression of the airway smooth muscle transcriptome in fatal asthma. PLoS One.

[4] Xystrakis E, Kusumakar S, Boswell S, Peek E, Urry Z, Richards DF (2006). Reversing the defective induction of IL-10-secreting regulatory T cells in glucocorticoid-resistant asthma patients. J Clin Invest.

[5] Brehm JM, Schuemann B, Fuhlbrigge AL, Hollis BW, Strunk RC, Zeiger RS (2010). Serum vitamin D levels and severe asthma exacerbations in the Childhood Asthma Management Program study. J Allergy Clin Immunol.

[6] Corren J, Parnes JR, Wang L, Mo M, Roseti SL, Griffiths JM (2017). Tezepelumab in adults with uncontrolled asthma. N Engl J Med.

[7] Yang, Han Z, Oppenheim JJ*.* Alarmins and Immunity. Immunol Rev. 2017;280:41–56. doi:10.1111/imr.12577. 10.1111/imr.12577 PMC5699517 29027222

[8] Wallrapp A, Riesenfeld SJ, Burkett PR, Abdulnour RE, Nyman J, Dionne D (2017). The neuropeptide NMU amplifies ILC2-driven allergic lung inflammation. Nature.

[9] Sinclair C, Bommakanti G, Gardinassi L, Loebbermann J, Johnson MJ, Hakimpour P (2017). mTOR regulates metabolic adaptation of APCs in the lung and controls the outcome of allergic inflammation. Science.

[10] Matthey M, Roberts R, Seidinger A, Simon A, Schröder R, Kuschak M (2017). Targeted inhibition of Gq signaling induces airway relaxation in mouse models of asthma. Sci Transl Med.

